# Evaluation of the effects of focused ultrasound stimulation on the central nervous system through a multiscale simulation approach

**DOI:** 10.3389/fbioe.2022.1034194

**Published:** 2022-12-01

**Authors:** Alessia Scarpelli, Mattia Stefano, Francesca Cordella, Loredana Zollo

**Affiliations:** Department of Engineering, Research Unit of Advanced Robotics and Human-Centred Technologies, Università Campus Bio-Medico di Roma, Roma, Italy

**Keywords:** transcranial focused ultrasound stimulation, computational modeling, multiscale simulation, sensory feedback, prosthetic systems

## Abstract

The lack of sensory feedback represents one of the main drawbacks of commercial upper limb prosthesis. Transcranial Focused Ultrasound Stimulation (tFUS) seems to be a valid non-invasive technique for restoring sensory feedback allowing to deliver acoustic energy to cortical sensory areas with high spatial resolution and depth penetration. This paper aims at studying in simulation the use of tFUS on cortical sensory areas to evaluate its effects in terms of latency ad firing rate of the cells response, for understanding if these parameters influence the safety and the efficacy of the stimulation. In this paper, in order to study the propagation of the ultrasound wave from the transducer to the cortical cells, a multiscale approach was implemented by building a macroscopic model, which estimates the pressure profile in a simplified 2D human head geometry, and coupling it with the SONIC microscale model, that describes the electrical behaviour of a cortical neuron. The influence of the stimulation parameters and of the skull thickness on the latency and the firing rate are evaluated and the obtained behaviour is linked to the sensory response obtained on human subjects. Results have shown that slight changes in the transducer position should not affect the efficacy of the stimulation; however, high skull thickness leads to lower cells activation. These results will be useful for evaluating safety and effectiveness of tFUS for sensory feedback in closed-loop prosthetic systems.

## 1 Introduction

One of the main limitations of commercial prostheses is the lack of sensory feedback to the user. However, in recent research studies, sensory information coming from the environment is detected by using sensorized prostheses, encoded by means of specific algorithms, and delivered to residual peripheral nerves of amputees through neural interfaces.

**FIGURE 1:**
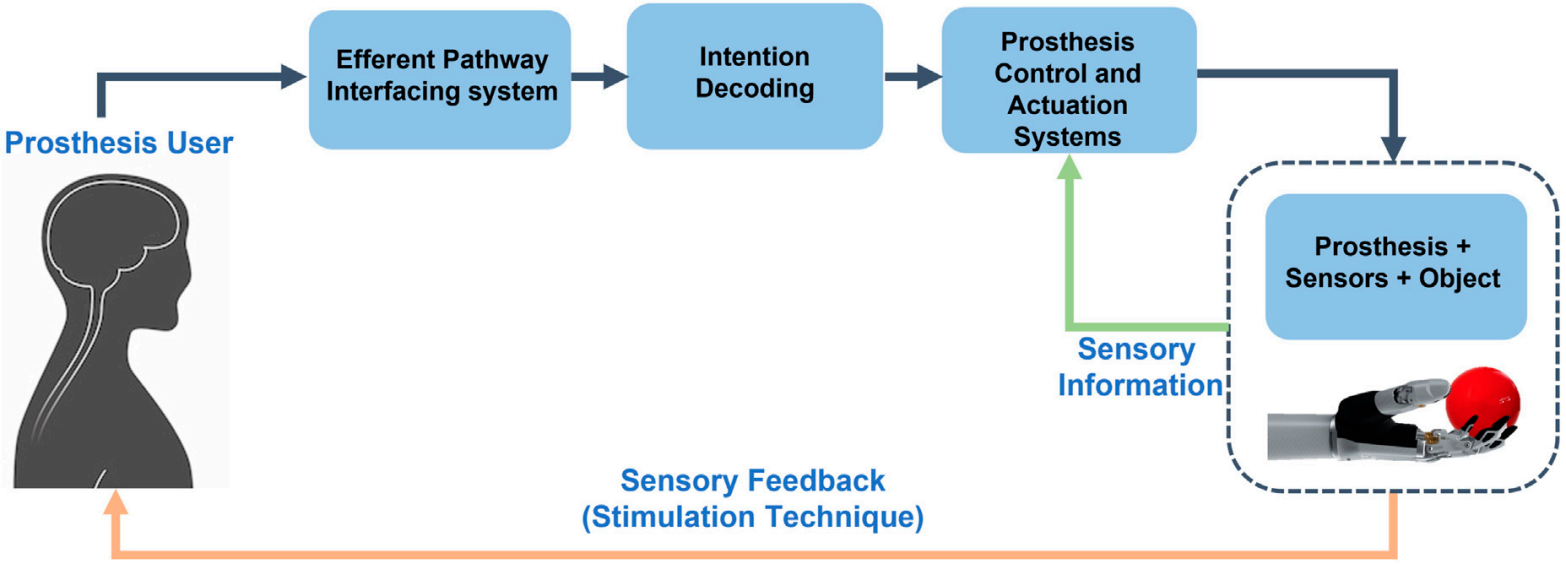
Block scheme of the control loop of a prosthetic system.

In Figure 1, a schematic view of the closed-loop control of a hand prosthesis with sensory feedback is shown. The bidirectional communication between the prosthesis and the user is reestablished by integrating the user in the control loop, through sensory feedback restoration. User intentions are captured by the interfacing system of the efferent pathway, then decoded and employed to control the prosthesis. While manipulating objects, external sensory information caught by the sensors arranged on the prosthesis, is processed and given back to the user by means of sensory feedback techniques.

Several stimulation techniques, applied to the Peripheral and the Central Nervous Systems, have been studied in literature to elicit sensory information in the most natural way as possible. So far, the best solution to guarantee high stimulation selectivity and spatial discrimination of the hand areas is based on invasive neural interfaces used to electrically stimulate the peripheral nerves (Raspopovic et al., 2014; Zollo et al., 2019; D’Anna et al., 2019; George et al., 2019). The main limitation of this solution is the necessity of a surgical intervention which strongly limits the clinical translation of this research. Therefore, over the years, researchers tried to find an alternative non-invasive solution able to deliver close-to-natural somatic sensations.

Traditional non-invasive brain stimulation techniques, such as Transcranial Magnetic Stimulation (TMS) and Transcranial Direct Current Stimulation (tDCS) (Fregni and Pascual-Leone, 2007; George and Aston-Jones, 2010) are generally used for neuromodulation for the treatment of brain diseases. Differently, recent studies on CNS showed that Transcranial Focused Ultrasound Stimulation (tFUS) seems to be a valid solution for brain stimulation because it demonstrated to be discriminatory and selective. In fact, with respect to the other techniques, which are able to stimulate cortical regions of the order of centimeters, tFUS can deliver acoustic energy to areas of the order of millimeters.

tFUS showed to be able to target both cortical and deeper brain regions. It allowed to stimulate human primary and secondary somatosensory cortex (Lee et al., 2016; Legon et al., 2018) eliciting different tactile sensations in different regions of the hand (Legon et al., 2014; Lee et al., 2015). In the study of (Lee et al., 2015), tFUS evoked transient somatic sensations on the hand and/or the fingers of 11 out of 12 healthy subjects. A perception of tingling was the most reported type of sensation, even though other qualities, like itching or heaviness have been perceived.

Through changing the acoustic intensity of the beam, tFUS showed to reach also deeper brain regions. For instance, at low intensities Subthalamic Nucleus (STN) neural activity was modulated with a safe and low intensity ultrasonic brain stimulation, without activating overlying cortical areas (Tarnaud et al., 2018).

More recently, some studies aimed at understanding the fundamental mechanism of interaction between tFUS and neural cells. A recent study showed that neuromodulation induced by tFUS affects both large scale target regions in the brain and connectivity within the small circuits. tFUS is capable of producing in the somatosensory cortex of rats local neural circuits correlation or decorrelation, which measure association or not between neuronal firing times, at different PRFs as well as excitation on an individual neuronal level (Ramachandran et al., 2022).

Focusing on the small scale regions, tFUS has shown to have an efficient neuromodulatory effect on brain circuits. Attention has been paid on understanding how tFUS interacts with different types of neurons. For example, fast-spiking (FS) and regular-spiking (RS) neurons response to tFUS application was deeply investigated: results have shown that they differently behave in response to different Pulse Repetition Frequency (PRF) of the FUS beam. FS demonstrated an inhibitory behaviour, whereas RS an excitatory by increasing the value of PRF from 30 to 4500 Hz (Yu et al., 2021). The most important achievement was the fact that in that way it is possible to specifically select the type of neuron by tuning the tFUS PRF.

Therefore, tFUS could represent a significant advancement in the field of interfaces for sensory feedback restoration in neuroprostheses, thanks to its non-invasive and selective features.

Although tFUS is a non-invasive method, attention has to be payed to the power intensity applied to the target region. In fact, if the power intensity is above a safety threshold, it can lead to a dangerous temperature increase in biological tissues (Pasquinelli et al., 2019). Therefore, the development of neurocomputational models, which describe the effect of tFUS on tissues, would be useful to predetermine the safest and most effective stimulation parameters.

In literature, studies have focused on simulating the macroscopic propagation of the ultrasound wave in different media, which resemble the properties of skull and/or cerebral matter (Mueller et al., 2016, 2017).

One of the main microscopic mathematical description was hypothesized in the Neuronal Intramembrane Cavitation Excitation (NICE) model (Krasovitski et al., 2011; Plaksin et al., 2014). Later, to computationally optimize the model, the SONIC model has been developed: it implements the same equations but guarantees lower computational time (Lemaire et al., 2019). These studies hypothesizes that ultrasound induces the cavitation of phospholipidic structures, named bilayer sonophore (BLS), within the membrane, which oscillates around the rest position because of the pressure stimulus. Variations in the membrane capacitance produce an electric neuron response.

Currently, macroscale, and microscale models have been studied in literature only separately. A multiscale analysis that permits to couple the biological tissues response with neural cell membrane displacement would be relevant.

Until now, the correlation between the electrical behaviour of neurons and the elicited sensation perceived by subjects who underwent to tFUS, has not been deeply investigated yet and there is no information about this relationship.

This paper aims at studying, by means of a multiscale simulation method, the tFUS effects onto the brain under different conditions. It will help to define the optimal stimulation settings for potentially eliciting tactile sensations in amputees and therefore improving the embodiment and the dexterity of the prosthetic hand. The electrical response of a single-point neuron to tFUS is obtained by coupling the estimated pressure profile in the brain, provided by the macroscopic wave propagation study developed in k-Wave, with the SONIC microscale model.

To correlate the sensations reported by human subjects, who underwent to tFUS (Lee et al., 2015), with the simulated neural behaviour, the stimulation parameters adopted in this paper resemble the ones used in (Lee et al., 2015), where FUS stimulation of the human somatosensory cortex is performed.

The proposed multiscale approach models a head with a scalp according to the anatomical properties of three subjects of the study with different skull thicknesses, i.e. 5.4, 7.6, and 10.5 mm, in order to obtain the electrical behaviour of the cortical neurons in specific locations.

## 2 Materials and methods

### 2.1 Macroscale model

In the macroscale simulations performed in k-wave, the ultrasound wave propagation applied by tFUS in a 2D simplified human head was evaluated. The ultrasound stimulation pattern was settled according to the acoustic parameters used to generate the pulsed FUS for the transcranial brain stimulation of the 12 participants of the study of Lee et al. (2015). As in the literature, the sinusoidal acoustic pressure waves were fixed at a frequency of 250 kHz, which is also the operating fundamental frequency of a commercial ultrasound transducer, i.e. H-115 Sonic Concepts transducer. Despite literature parameters, for which each batch of the pulsed stimulation consisted of a 1 ms burst, in this work the maximum simulation duration consisted of 500 *μ*s (DC of 100%), and the minimum duration of the pulse stimulus was fixed at 200 *μ*s (DC of 40%). This values were above the minimum duration *t*_*ss*_ (190 *μ*s) to reach a steady-state. *t*_*ss*_ was obtained as follows

$$t_{ss}=\frac{\sqrt{\left(s_{x}^{2}+s_{y}^{2}\right)}}{\nu_{s}}$$ (1)

where x and y are the grid lengths in the *x* and *y* direction, respectively, and *ν*_*s*_ is the minimum sound velocity in the medium evaluated between the considered media.

**FIGURE 2:**
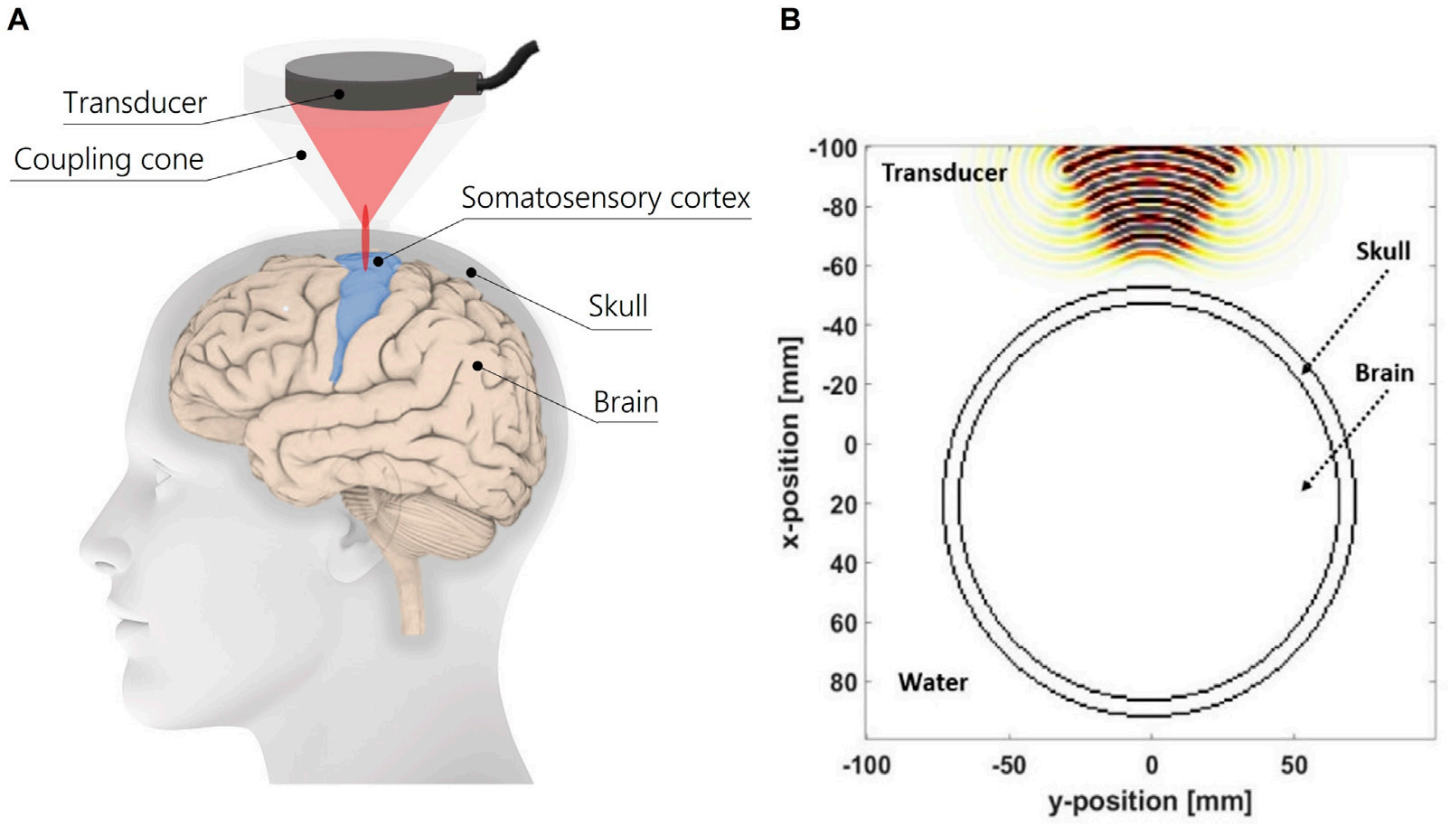
**(A)** Schematics of the tFUS setup, **(B)** geometry of the 2D simplified human head model.

In Figure 2A is represented a schematics of the experimental setup for tFUS on the somatosensory cortex for eliciting tactile sensations on upper limb amputees. The modelled geometry of the head is composed of a skull layer, brain tissue and a coupling medium, i.e., water. The diameter of simplified head model is considered according to Salkim et al. (2019). Moreover, the profile of the ultrasound transducer is reproduced based on the H-115 transducer, which is comparable to the one used on humans in the study of (Lee et al., 2015). In Figure 2B the modelled geometry of the model is reported. For each biological tissue, the physical properties of each medium are listed in Table 1.

**TABLE 1:** Media properties.

	c_*s*_[*m*/*s* ^2^]	*ρ* [kg/m^3^]	*α*_0_[*dB*/*MHz* ⋅ *cm*]
Water Treeby and Cox (2014)	1481	998	0.002
Skull Treeby and Cox (2014)	2820	1732	7.75
Brain Baron et al. (2009)	1500	1000	0.8

*c*_*s*_, sound velocity in medium; *ρ*, density of the medium; *α*_0_, medium constant to evaluate the absorption coefficient.

Two concentric circles modeled the brain and skull regions: the inner region represented the brain, whereas, the properties of the skull were assigned to the ring area. Three different values of skull thickness were considered: 5.4, 7.6, and 10.5 mm. The three values were chosen on the basis of the real skull thickness of three of the twelve healthy subjects who took part in the study on somatosensory cortex stimulation in (Lee et al., 2015). The three subjects were identified among the others because they experienced three different sensations when the tFUS protocol was administered: the subject with 5.4 mm of skull thickness reported to perceive tactile sensations on both hands, elbow, wrist, arm and forearm; the subject with the skull of 7.6 mm reported to feel sensations on both hands; whereas, the one with a skull thickness of 10.5 mm did not referred any sensation.

A coupling medium between the transducer profile and the skull layer was considered to reproduce the function of a coupling cone filled with degassed water. A distance of 4 cm was settled between the profile of the transducer and the outer border of the skull layer. This value was chosen on the basis of the dimensions of a commercial coupling cone C-103 of Sonic Concepts, which suited for the selected transducer H-115. This distance will also permit to target the somatosensory cortex region with the ultrasound focus.

Free water simulations were performed in order to identify the value of the ultrasound pressure amplitude for obtaining the intended amount of acoustic energy. 3 W/cm^2^ was chosen as safety threshold for the maximum acoustic intensity because it is in compliance with the international electrotechnical commission (IEC) 60601 part 2 standard for physiotherapy equipment. Moreover, an acoustic intensity of 3 W/cm^2^ demonstrated to be a sufficient level of energy for successful tFUS of the somatosensory cortex (Lee et al., 2015). By means of free water simulations, pressure amplitude was settled at 100 kPa (Plaksin et al., 2014; Lemaire et al., 2019) in order to obtain a maximum average intensity under the safety threshold. Considering that stimulation parameters, the maximum pressure and average intensity obtained into the model are under the safety threshold. The simulation environment consisted in the k-wave Matlab Toolbox, a k-space pseudospectral method-based solver (Treeby et al., 2012), employed to get the maximum pressure amplitude and average acoustic intensity maps. The simulations through the coupling medium (water), skull and brain layers were performed for six values of Duty Cycle (DC), from 40% to 90% with a step of 10%.

### 2.2 Microscale model

The SONIC model (Lemaire et al., 2019) based on the intramembrane cavitation neuronal BLS model (NBLS) of Plaksin et al., 2014 (Plaksin et al., 2014), was implemented to characterize the behaviour of Regular Spiking (RS) cortical neurons when tFUS is applied. This model coupled the modified BLS model (Krasovitski et al., 2011) and the Hodgkin and Huxley model (Pospischil et al., 2008), without re-tuning or post-hoc adjustments. These models were previously fitted on experimental data and the NBLS model predictions were compared to the *in-vivo* experiments on tFUS neurostimulation of the motor cortex of mice (King et al., 2013). Computational results demonstrated to be qualitatively in agreement with these experimental trials. However, in these early experimental data, the role of auditory confounding factors on the neural responses, could not have been deeply investigated. Experimental data has to be free from these artifacts, in order to guarantee that neural response is only due to targeted area stimulation (Guo et al., 2018; Sato et al., 2018). The authors of the study King et al., 2013 uncoupled the transducer from the mice’s heads and performed the tFUS. Although the authors asserted that suprasonic auditory responses, as a cause of sonication-evoked contractions, could be ruled out, this condition may have not been completely controlled.

In the microscale model, the modified Rayleigh-Plesset bubble cavitation equation Eq. 2 describes the variation of the deflection of the membrane (Z).

$$\begin{array}{c} \frac{d^{2}Z}{dt^{2}}+\frac{3}{2R\left(Z\right)}{\left(\frac{dZ}{dt}\right)}^{2}=\frac{1}{\rho_{l}R\left(Z\right)}\left[P_{in}+P_{M}-P_{0}+-P_{s}\left(Z\right)+P_{ec}+P_{A}\sin\left(\omega t\right)+\frac{4}{R\left(Z\right)}\frac{dZ}{dt}\left(\frac{3\delta_{0}\mu_{s}}{R\left(Z\right)}+\mu_{l}\right)\right] \end{array}$$ (2)

$$\frac{dn_{g}}{dt}=\frac{2S\left(Z\right)D_{gl}}{\xi }\left(C_{g}-\frac{P_{in}\left(Z\right)}{k_{H}}\right)$$ (3)

The pressure term *P*_*A*_sin(*ω*t) represents the ultrasound external stimulus, in which *P*_*A*_ is the pressure amplitude of the ultrasound. In the governing equation Eq. 2, other pressure terms contribute to model the underling mechanism of ultrasound brain stimulation:

• *P*_*in*_ is the internal pressure in the membrane $\left(P_{in}\left(Z,n_{g}\right)=\frac{n_{g}R_{g}T}{V\left(Z\right)}\right)$ ;
• *P*_*M*_ is the intermolecular pressure between leaflets ( $P_{M}\left(Z\right)=\frac{1}{S\left(Z\right)}\int_{0}^{2\pi }\int_{0}^{a}A_{r}\left(\gamma^{x}-\gamma^{y}\right)drd\theta$ with $\gamma =\frac{\Delta^{*}}{2z\left(r\right)+\Delta \left(Q_{m0}\right)}$ );
• *P*_0_ is the constant pressure around the membrane;
• *P*_*s*_ is the membrane tension pressure in the two leaflets $\left(P_{s}\left(Z\right)=-\frac{k_{s}}{R\left(Z\right)}\frac{S\left(Z\right)-S_{0}}{S_{0}}\right)$ ;
• *P*_*ec*_ is the electric pressure generated on the *sonophore* by charges $\left(P_{ec}\left(Z,Q_{m}\right)=-\frac{S_{0}}{S}\frac{Q_{m}^{2}}{2\epsilon_{0}\epsilon_{r}}\right)$ .

In Eq. 2 R(Z) is the radius of curvature, *ρ*_*l*_ is the density of the extramembrane medium, *δ*_0_ is the thickness of the leaflet and *μ*_*l*_ and *μ*_*s*_ are respectively the viscosity of the extramembrane medium and of the leaflets.

The behaviour is also depending on the variation of the gas content in the membrane of the cell. Internal gas content equation is reported in Eq. 3.

In Eq. 3, *P*_*in*_ is the internal gas pressure in the cavity, S(Z) is the surface area of a leaflet, *ξ* boundary thickness for gas transport, *D*_*gl*_ is the diffusion coefficient, *C*_*g*_ is the gas concentration in the extramembrane medium and *k*_*H*_ is the Henry’s constant. All the parameters in Eqs 2, 3 were defined in (Plaksin et al., 2014; Lemaire et al., 2019). What activates the *V*_*m*_ transmembrane potential to oscillate are the periodic oscillations of the membrane capacitance, which varies as in Eq. 4 Krasovitski et al. (2011).

$$C_{m}\left(Z\right)=\frac{C_{m0}\Delta }{a^{2}}\left[Z+\frac{a^{2}-Z^{2}-Z\Delta }{2Z}\ln\left(\frac{2Z+\Delta }{\Delta }\right)\right]$$ (4)

Therefore the evolution of the membrane potential *V*_*m*_ was modified as it follows in Eq. 5:

$$\frac{dV_{m}}{dt}=-\frac{1}{C_{m}}\left[V_{m}*\frac{dC_{m}}{dt}+\underset{i}{\sum }G_{i}\left(V_{m}-V_{i}\right)\right]$$ (5)

However, the whole system has been recasted with the aim of optimizing the computational time of the neuron model, by solving the electrical fundamental equation in function of the charge, using the transformation *Q*_*m*_ = *C*_*m*_ ⋅ *V*_*m*_. The new fundamental equations, which depends on ionic currents by conductances *G*_*i*_ and potentials *V*_*i*_, are described in Eq. 6:

$$\left\{\begin{array}{c} \frac{dQ_{m}}{dt}=-\underset{i}{\sum }G_{i}\left(V_{m}^{*}-V_{i}\right)\;\\ \frac{dm}{dt}=\alpha_{m}^{*}\left(1-m\right)-\beta_{m}^{*}m\;\\ \vdots \;\\ \frac{dp}{dt}=\alpha_{p}^{*}\left(1-p\right)-\beta_{p}^{*}p\; \end{array}\right.$$ (6)

m, h, n, p are the gating variables respectively of sodium (m and h), potassium (n) and slow non-inactivating (p). $\alpha_{x}^{*}$ and $\beta_{x}^{*}$ are the specific voltage-dependent activation and inactivation rate constants. Moreover, Eq. 6 was calculated implementing an hybrid approach, as a function of “effective” variables, denominated with the superscript “*”, which were obtained by averaging their behaviour over the acoustic period T.

In Table 2 parameters of the microscale model are shown. The SONIC model was implemented in Matlab environment.

**TABLE 2:** Parameters of the model.

Parameter	Symbol	Value
Sonophore radius	a	32.0 *nm*
Temperature	T	309.15 *K*
Universal Gas Content	R_*g*_	8.314 *Pa* ⋅ *m* ^3^ ⋅ *mol* ^−1^ ⋅ *K* ^−1^
Intermolecular pressure coefficient	A_*r*_	10^5^ Pa
Intermolecular repulsion term	x	5
Intermolecular attraction term	y	3.3
Space between leaflets	Δ*	1.4 *nm*
Extramembrane pressure	P_0_	10^5^ Pa
Area compression modulus of the membrane	k_*s*_	0.24 *N* ⋅ *m* ^−1^
Vacuum permittivity	*ϵ*_0_	8.854 10^−12^ F m^−1^
Relative permittivity	*ϵ*_*r*_	1
Density of the extramembrane medium	*ρ*_*l*_	1075 *kg* ⋅ *m* ^−3^
Viscosity of the extramembrane medium	*μ*_*l*_	7 ∗ 10^−4^ Pa ⋅ *s*
Viscosity of the leaflets	*μ*_*s*_	0.035 *Pa* ⋅ *s*
Thickness of the leaflets	*δ*_0_	2 *nm*
Diffusion coefficient of air	D_*gl*_	3.6 ∗ 10^−9^ m^2^ ⋅ *s* ^−1^
Effective thickness of boundary layer for gas transport	*ξ*	0.5 *nm*
Resting membrane capacitance	C_*m*0_	1 *μF* ⋅ *cm* ^−2^
Henry’s constant	k_*H*_	1.013 *Pa* ⋅ *m* ^3^ ⋅ *mol*

### 2.3 Multiscale approach

**FIGURE 3:**
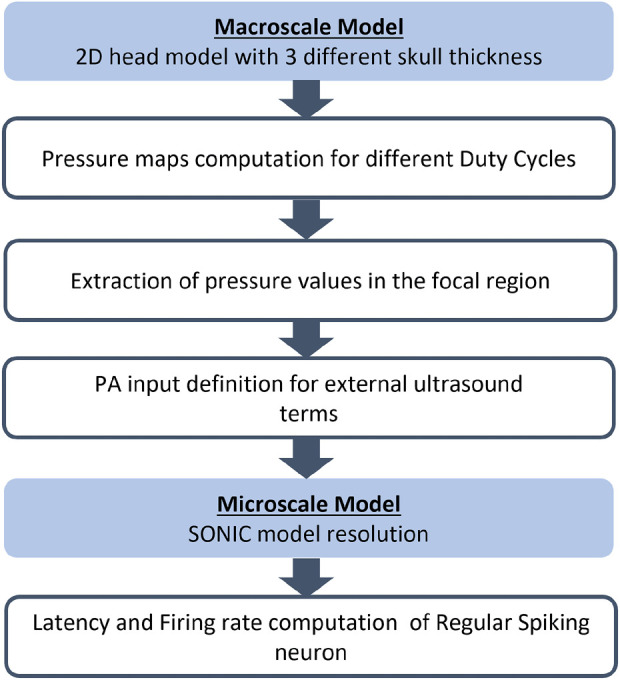
Flowchart of the proposed multiscale approach for each simulation with human head geometry with the three different skull thickness (5.4, 7.6, and 10.5 mm).

The flowchart of the proposed approach for the multiscale analysis of the effects of ultrasound is shown in Figure 3. In the following section, the detailed description of the proposed approach is reported.

**FIGURE 4:**
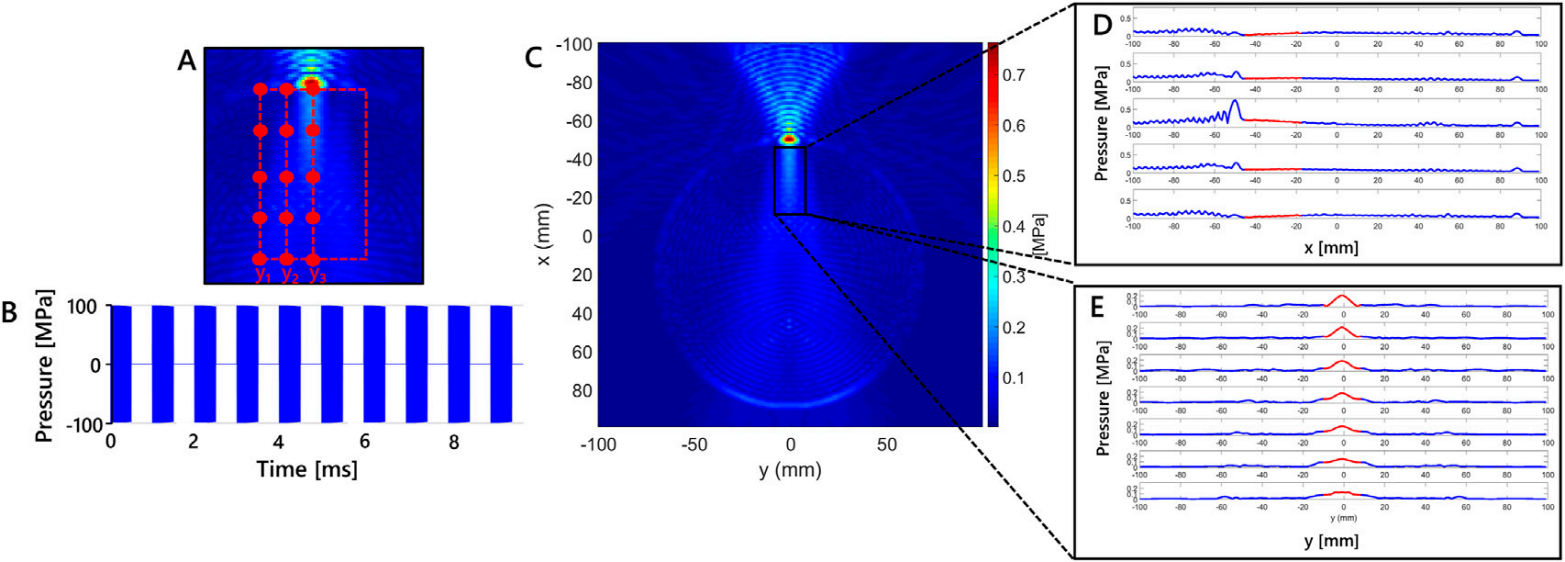
Macroscale model results showing a 2D grid color map of maximum pressure due to the tFUS stimulation for modelled skull thickness of 5.4 mm. **(A)** Enlargement of the region of interest. The 15 points selected considering three different y-coordinates (i.e., *y*_1_ = −10.19, *y*_2_ = −5.56 and *y*_3_ = −0.92 mm) in simulation at 5.4 mm of skull thickness are shown. **(B)** Schematics of the stimulation waveform used in the simulation study. Each 1 ms burst of sonication pulses operating at a pulse repetition frequency of 500 Hz is a sinusoidal wave at 250 kHz. **(C)** Maximum pressure (MPa) in x-y plane for the simulation at 5.4 mm, **(D)** Maximum pressure variation along x for five fixed values of y-coordinate, red line highlights the region of interest along x. **(E)** Maximum pressure variation along y for seven fixed values of x-coordinate, red line indicates highlights the region of interest along y.

From the macroscale model, a first simulation was performed using the value of 5.4 mm for the skull thickness in order to extract the necessary information from the output field maps. From the observation of pressure maps within the target area of the ultrasound stimulation, i.e. the focal region, 15 pressure values were extracted in 15 positions, as detailed in Section 3. The points were identified in a specific brain area under the skull layer on the pressure map: the width of the region along the y-coordinate was selected considering the width of the focus; whereas the length along x-coordinate is decided on the basis of the reported focal dimensions from the transducer datasheet, which is 36 mm. Taking into account the symmetry of the macroscale simulation, the 15 pressure values were identified along the transducer axis and on the left side of the area. In that region 5 points were extracted for three different y-coordinates. In Figure 4A, the three y-coordinates are identified by the *y*_1_, *y*_2_ and *y*_3_ positions.

For each simulation, the identified points in the region of interest were used as pressure input to the SONIC model for each value of the stimulus DC. It is assumed that a single-point neuron could be located in each point considered, to evaluate if displacement from the center of the focal region can lead to neuron activation. So we used single-point neurons as a probe to evaluate the efficacy of the stimulation. The selected pressure values were used as pressure amplitude inputs (*P*_*A*_) to the ultrasound external pressure term (*P*_*A*_sin(*ω*t)) described by a modified Rayleigh-Plesset equation (Eq. 2). In the model the stimulation duration of the external pulsed ultrasound source was settled to 100 ms.

By means of the microscale simulations, the mechanical and the electrical behaviour of neurons were calculated and characteristic parameters were extrapolated: for each macroscopic simulation for each of the positions on the grid, the latency of the stimulus and the firing rate were computed. Latency is defined as the delay between the stimulus onset and the occurrence of the first spike. Firing rate is the average of reciprocals of inter-spike intervals of spikes occurring during stimulation (Lemaire et al., 2019).

## 3 Results

In Figure 4B the stimulation waveform used in the study is reported. Maximum pressure results in x-y plane for the simulation at 5.4 mm are shown in Figure 4C. The maximum values are observed in the region with skull layer. Maximum pressure values are higher in that region because of the distance from the transducer and different density properties of skull layer. That values are obtained using stimulation parameters that lead to intensity values below the safety threshold in free water, as reported in Section 2. Below it, the region delimited by the black rectangle in Figure 4C, is considered for the study because it is where location of cortical neurons is hypothesized. The dimension of the x-y region has been defined according to the cortical depth where the transducer focus can be found. The maximum pressure variation along x is shown in Figure 4D for five fixed values of y-coordinate, in which the red segments highlight the pressure variation in the rectangular region of interest for x direction. The maximum pressure variation along y is reported in Figure 4E for seven fixed values of x-coordinate. Similar to Figure 4D, the red line highlights the region of interest along y. The pressure variations of this set of fixed points is chosen to appreciate the maximum pressure variation.

**FIGURE 5:**
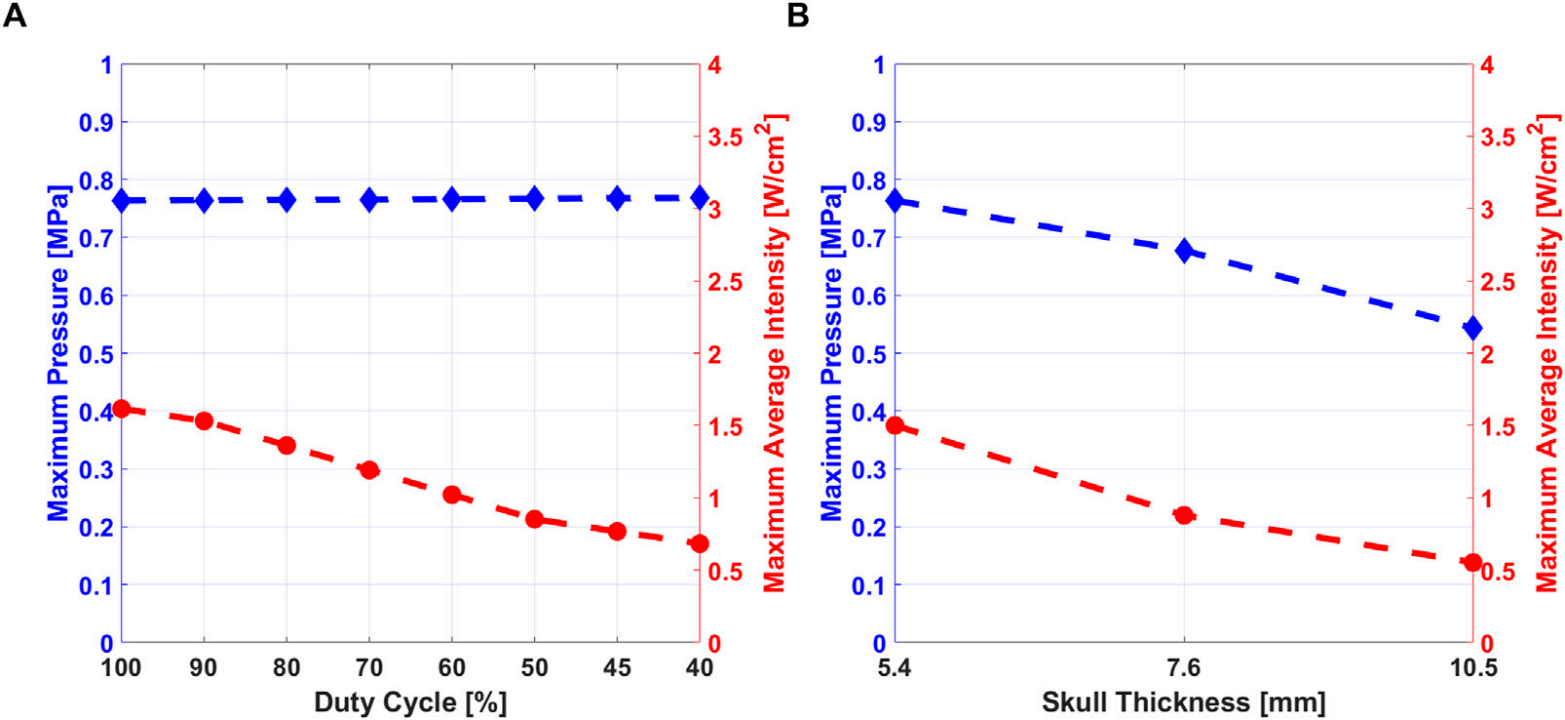
**(A)** Maximum pressure (blue) and Maximum Average Intensity (red) variation at different DC, for skull thickness of 5.4 mm. **(B)** Maximum pressure and Maximum average intensity variation at the three different values of skull thickness at DC = 90%.

The Maximum Pressure and the Maximum Average Intensity are shown in Figure 5A for the simulation at 5.4 mm at different DC. The maximum values reached in biological media are considered to be compared with safety thresholds defined in literature. The maximum pressure and the average intensity for the three different skull thickness considered in the study for the simulation at 500 *μ*s (DC = 90%) are shown in Figure 5B.

**FIGURE 6:**
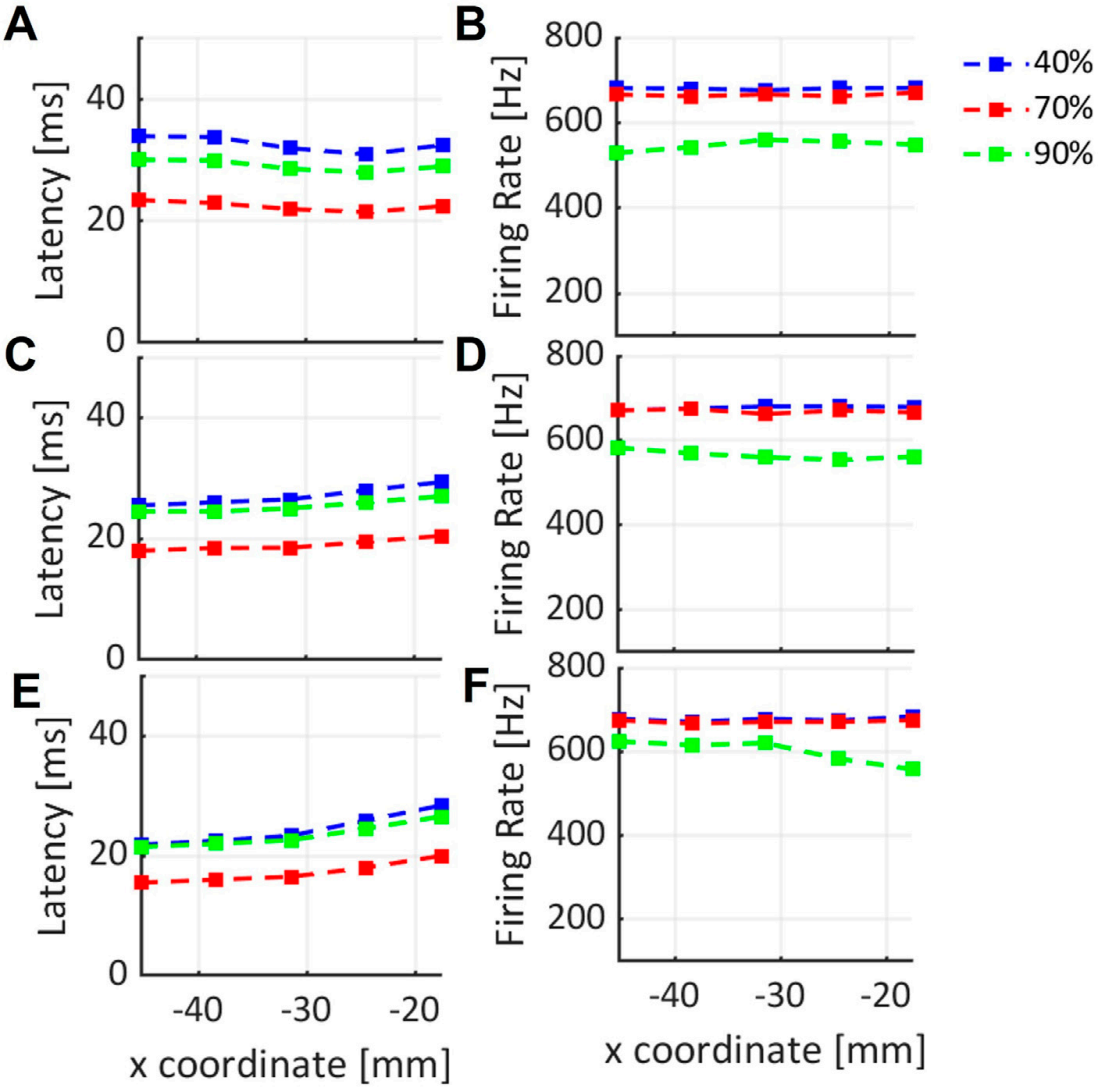
Variation of latency and firing rate of neural cell response to the ultrasound stimulus along x-coordinate, for three different y-coordinates: **(A–F)** are related to *y*_1_, *y*_2_, *y*_3_ values respectively, selected in the region of interest for three DC values shown in blue, red and green lines.

The latency and the firing rate variation along x-coordinate, are shown in Figure 6 for three different values of DC stimulus. The results were obtained for simulations with skull thickness at 5.4 mm. Figures 6A,B are related to y-coordinate *y*_1_, Figures 6C–F are related to *y*_2_ and *y*_3_ values.

**FIGURE 7:**
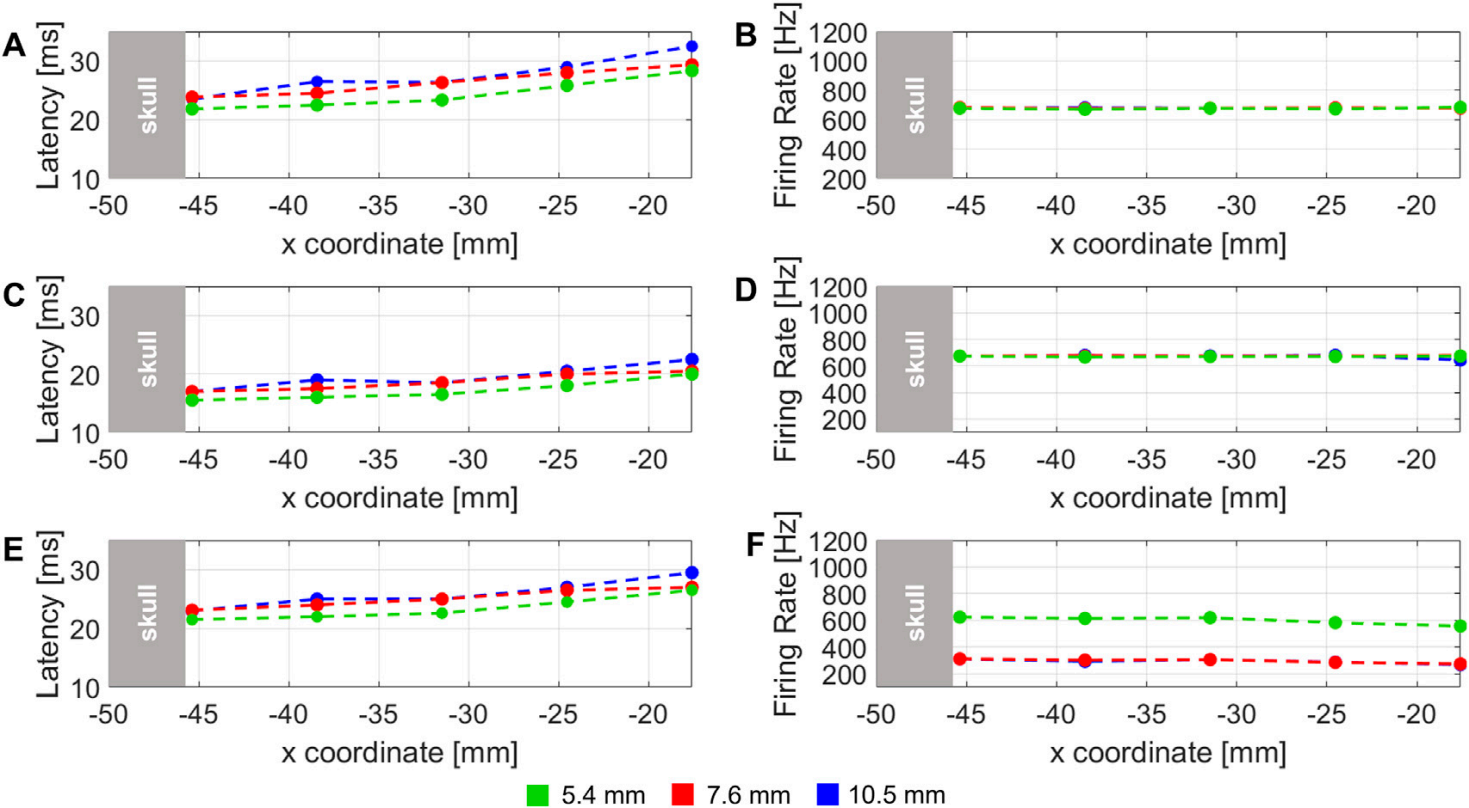
Latency of the stimulus and firing rate computed for three different DC: **(A–F)** are related to 40% DC, 60%, and 90% DC stimulus, respectively. The dashed lines represent the three different skull thickness considered of 5.4 (green), 7.6 (red) and 10.5 mm (blue).

Similarly, latency and firing rate of neural cell electrical response along x-coordinate, in the axial direction of the transducer (*y*_3_), are shown in Figure 7. Latency and firing rate were computed for the three different values of skull thickness, that were considered in the study of (Lee et al., 2015) (Lee et al., 2015).

## 4 Discussion

Maximum pressure amplitude in the region highlighted in Figure 4C, was studied at different values of DC. The maximum pressure variation is shown in the region under the skull that has been considered to evaluate the pressure values used in RS mathematical model.Pressure stimulus and its duration are related to the generation of action potential by a neural cell, as discussed in (Plaksin et al., 2014). Therefore, pressure amplitude can be considered as an index of stimulation efficacy. From the results shown in Figure 5, maximum pressure amplitude and maximum average intensity were studied. As cited in the previous sections, the maximum average intensity values into the biological tissues have to be under safety threshold values. The comparison between the maximum pressure values and the maximum average intensity observed from the macroscale simulation, allows concluding that lower values of DC are related to lower values of average intensity, i.e. power delivered to tissues. As Figure 5A shows, pressure amplitude was not affected by different DC values, so the efficacy of the stimulation does not depend from DC. A stimulus with low DC can be safer and effective than another one with high value of DC (Kim et al., 2014).

In Figure 5B, it is possible to observe that when the skull thickness increases, both pressure and average intensities values decrease, because the ultrasound stimulus is more shielded by the skull. Comparing obtained results with experimental data, the three subjects considered in this study, reported different areas of the sensations (Lee et al., 2015): by analysing the elicited regions for each subjects, it could be possible to correlate the sensations with their skull thickness and therefore with the value of acoustic pressure/intensity delivered at the target. The subject with 10.5 mm of skull thickness was the only participant involved in the study who did not perceived any tactile sensations: the authors of the experimental work hypothesized that the reason was that US beam was more attenuated due to the skull thickness outside of standard values. In our study, the results obtained from simulations confirmed that hypothesis: high skull thickness is related to lower pressure and intensity into the brain, so the stimulation would be less effective. From these results it is possible to suggest an optimal stimulation pressure range for which the subjects would report the intended perceptions. At 7.6 mm sensations were elicited only on the hand, properly excluding non target regions (arm ,wrist, forearm and elbow), as differently happened for the subject with 5.4 mm of skull thickness. Therefore, taking into consideration the fact that skull thickness is an intrinsic property of each subject that cannot be changed, it is feasible to modulate maximum pressure at the target, which should be less than ∼0.8 MPa and more than ∼0.55 MPa.

In Figure 6 the results of the simulation at 5.4 mm are reported. Latency variation along x-coordinate for three fixed value of y-coordinate, *y*_1_ = –10.19, *y*_2_ = –5.56 and *y*_3_ = –0.92 mm, respectively, are shown in Figures 6A–E. In each plot, different latency values according to the three considered DC, i.e. 40%, 60% and 90%, can be observed. From these results, when *y*_1_ is fixed (Figure 6A), the latency can be considered globally constant along x-coordinate in the considered range of 20 mm, for each value of DC. An increase in latency is shown in Figures 6C–E along the x-coordinate, for each DC value. This result can be related to the corresponding decreasing maximum pressure values found at *y*_2_ and *y*_3_ along x-axis in the focal region under the skull (Figure 4B). The results in Figures 6B–F show that the firing rate of RS neuron is globally constant along x-direction for the three different DC values. In particular, a small decrease for the firing rate when 90% of DC is used is evident from Figure 6F. It can be related to a corresponding decrease of pressure amplitude along x-axis. A different behavior is observed from the trends related to 40% and 70% DC, where a globally constant value is observed along x.

The latency variation related to three skull thicknesses along x-coordinate at *y*_3_ fixed (i.e. along the symmetry axis of transducer) is shown in Figures 7A–E for 40%, 60% and 90% DC, respectively. In all the plots, a global increase of latency can be observed for all the thicknesses and all the DCs. This behaviour, similarly to the results obtained in the analogue case in Figure 6, can be related to a decrease of pressure amplitude along x. It is also possible to note that, in each plot, at each x-value, latency values decrease according to skull thickness. This is a reasonable results because it is expected that using the same stimulus, for a lower skull thickness, generates higher values of pressure amplitude below the skull, because the stimulus is less shielded from the skull if compared with an higher skull thickness.

Firing rate variation, shown in Figures 7B–F, is globally constant along the x-coordinate in the 20 mm range considered, for the three different skull thickness values. Only from Figures 7A,F small decrease related to 90% DC is evident for 7.6 and 10.5 mm, probably due to a corresponding decrease of pressure amplitude, observed yet as a consequence of the corresponding latency variation in the same locations. A comparison of firing rate among different skull thicknesses can be also considered. Figures 7B–D shows that all trends are overlapped along x. Only in Figure 7F, the trend related to 5.4 mm of skull thickness is at higher firing rate values. It can be due to less shielding from the skull and therefore higher pressure values observed in the studied locations.

These results about different skull thicknesses can be compared with the hypotheses in (Lee et al., 2015) related to no perceived sensations in subject with 10.5 mm of skull thickness. From the simulation results in Figure 7, a lower trend of firing rate is observed compared with the 5.4 mm thickness value. So from our results, considering a mathematical model of RS point neuron, different values of firing rate and latency are observed when skull thickness of 10.5 mm is used. Therefore, the neuron is considered activated when the three thicknesses are used. However, a similar behaviour is obtained for both 7.6 and 10.5 mm at 90% of DC. For these values of skull in the experimental trial subjects reported to feel opposite sensations. This suggests that pressure amplitude at the target would not be the only crucial factor of stimulation efficacy. A possible reason could be the value of average intensity, so the amount of energy delivered in the specific area: for instance, the geometrical properties of the acoustic focus could be altered because of the passage through the highest skull thickness (10.5 mm), so stimulating a smaller portion of S1 cortex. Otherwise, the overall 2D simplification about the geometry of the human head, could have not taken into account of subject-specific features, which consequently have been neglected by this approximation. More detailed studies are needed to understand which is the main reason that correlates neuron activation to tactile sensations elicited in human subjects and to consider different neurons instead of a single-point one to study their global activity.

The obtained results from the multiscale analysis in simulation allowed us to investigate the tFUS effects on cortical neurons and its relationship with tactile sensations. It represents a fundamental step for closed loop prosthesis leading to limit preliminary experiments on animals and humans. In fact, neuron activation and response can be evaluated for different stimulus properties and the suitable parameters for effective stimulation can be obtained.

The employment of this multiscale approach for neurocomputational modelling would be fundamental for better investigating how tFUS stimulation interacts with neural structures at different levels. Various stimulation patterns could be tested in the multiscale analysis, so as to identify the behaviour of a single-point neuron when stimulated with the novel sensory feedback technique. Therefore, the experimental trials on humans can be designed on the basis of the results obtained from the simulations. The suitable stimulation parameters need to be set according to human head thickness for each subject. The estimation of the most suitable stimulation parameters would be a valuable tool for the design of novel interface technologies, such as miniaturized ultrasound transducers, and for placement optimization of these probes for multichannel stimulation. As a consequence, the stimulation waveform, able to elicit tactile sensations in upper limb prosthesis users, can be predetermined in simulation.

## 5 Conclusion

Several techniques have been developed to restore the bidirectional communication between upper limb prostheses and the users. The development of novel interfacing technologies needs to be simultaneous to the creation of efficient methods for evaluating, as a first step, their effects on biological entities. Neurocomputational models provide the literature with advantageous tools for controlling the efficiency and the safety of the technology.

In this paper, a multiscale approach for studying the effects of tFUS on central neural structure has been developed and the relationship between stimulation parameters and human physical characteristics with tactile sensory feedback in amputees has been studied. The developed macroscale model of wave propagation into tissues was coupled with a microscale model on neural behaviour of cortical neurons to study in simulation how the ultrasound beam propagates from the transducer to the cortical cells. In that way, it is possible to estimate which is the real intensity delivered at the target that practically acts on the neurons. tFUS is applied to a simplified 2D model of the human head developed in k-wave Matlab Toolbox, with stimulation parameters similar to the ones employed in the study of Lee et al. (2015), where FUS was applied to the somatosensory cortex of 12 healthy subjects. Three different k-wave simulations were performed varying the value of the skull thickness in the model geometry: 5.4, 7.6 and 10.5 mm. A set of pressure amplitude values were selected on the pressure maps at specific positions and used as inputs for the simulations of the microscale model.

The three macroscale simulations have shown that the efficacy of the stimulation, in terms of pressure, remained stable across the different DC (Stefano et al., 2020); in addition, the average acoustic intensity resulted in lower values for lower DC, thus demonstrating that the safety is proportionally related to low DC. Moreover, pressure values and acoustic intensities decreased with higher skull thicknesses.

The results about the computation of latency and firing rates showed that across the y-coordinates slight changes in position of the transducer should not affect the efficacy of the stimulation. Moreover, considering that there were no significant variations among DC in terms of both variables, a lower value of DC should be chosen to deliver less acoustic energy, continuing to guarantee the effectiveness of the stimulation.

Results were compared with the hypotheses in Lee et al. (2015), according to which the subject did not perceive sensations because the skull thickness is outside of the means. Certainly, high skull thickness is related to lower pressure and intensity into the brain, leading to the hypotheses that the stimulation would be less effective. However, the neuron responded to the three thicknesses. Hence, more detailed studies are needed to understand which is the main reason that correlates neuron activation to tactile sensations elicited in human subjects.

Future work will be devoted to: 1) improve the analysis of the effects of tFUS on the human head by increasing the complexity of the macroscale and the microscale models, for example considering other kind of cortical neurons; 2) take into account a more realistic geometry of the tissues of the human head. Moreover, an improvement of the SONIC microscale model would be the extension of the model from single-point model to a multicompartimental one, in order to account for a more realistic organization of the biological system.

## Data Availability

The raw data supporting the conclusions of this article will be made available by the authors, without undue reservation.
